# Monitoring the Whole Cycle Length Change of Cement Mortar Incorporated with SRA by CMOS Image Sensor

**DOI:** 10.3390/s20020468

**Published:** 2020-01-14

**Authors:** Hao Wu, Yan Yao, Ling Wang, Ruijun Gao, Shuang Lu

**Affiliations:** 1 China Building Materials Academy, Beijing 100024, China; wuhao1@cbmamail.com.cn (H.W.); yaoyan@cbmamail.com.cn (Y.Y.); wangling@cbmamail.com.cn (L.W.); gao_423@163.com (R.G.); 2 State Key Laboratory of Green Building Materials, Beijing 100024, China; 3 School of Civil Engineering, Harbin Institute of Technology, Harbin 150001, China; 4 Key Lab of Structures Dynamic Behaviour and Control of the Ministry of Education, Harbin Institute of Technology, Harbin 150090, China

**Keywords:** shrinkage-reducing admixture, length change, CMOS sensor, polymerization state, cement mortars

## Abstract

This paper introduces a new method to measure whole cycle length change non-destructively and continuously using a digital image analysis system. The macroscale length changes of mortars containing different shrinkage-reducing admixture (SRA) dosages (0%, 1%, 2% and 5% by cement weight) were first determined using a complementary metal oxide semiconductor (CMOS) image sensor under alternating dry and wet curing conditions. After that, the length change was calculated using developed digital image processing technology (DIPT) software. After that, several significant conclusions could be drawn by combining with the results of systematic tests of the macroscopic and microscale physical properties of the cement mortar using X-ray diffraction, scanning electron microscopy, mercury intrusion porosimetry (MIP) and nuclear magnetic resonance (NMR) methods. The test results indicated that SRAs exhibited significant effects on the shrinkage inhibition of cement mortars, whereas the shrinkage reduction behaviour was also affected by varying the curing conditions. The MIP and NMR analyses demonstrated that SRAs reduced the irreversible shrinkage of the cement mortars by decreasing the volume percentage of the 3–50 nm pores and promoting the conversion of calcium silicate hydrate gel from an oligomeric to a high polymerization state thereby improving the volume stability of cement mortars.

## 1. Introduction

Current civil engineering technology advancements have enabled rapid global urbanization and overall improvements in lifestyles which have resulted in cementitious materials being the most widely used and successful building materials. However, their unsatisfactory volume stability, including their unrestrained shrinkage or shrinkage–cracking under restrained conditions, have raised many safety and economic concerns such as crack generation accelerating the damage of concrete and thereby shortening the service life of structures. In addition, the usage of a high-strength and low-water-to-cement (W/C) ratio concrete has become more prevalent, and therefore, the dimensional stability for prolonging the service life of concrete structures has been emphasised [1]. Moreover, several specific high-performance additives have been developed to improve the volume stability of concrete such as expansive agents [2,3], fibres [4,5] and shrinkage reducing admixtures (SRAs) [6]. The shrinkage compensating mechanisms of ettringite (alumina, ferric oxide and tri-sulphate (AFt) phase), a commonly used expansive agent, have not been entirely clarified yet, and there are no guarantees that the manufacturing and subsequent expansion of concrete would ever be desirably controlled even if adequate curing was performed early on [2,7,8,9]. Nevertheless, the addition of AFt increases the cost and deadweight of concrete. The reason for the expansion properties of MgO- and CaO-type expansive agents varying with different calcining temperatures and residence times has not been clearly demonstrated [10]. Therefore, SRAs could be good additive options, as they also exert excellent early shrinkage retardation effects on concrete.

Several studies have demonstrated that the addition of SRAs is a reliable method for reducing autogenous [7,10,11] and plastic shrinkage [12,13,14,15] and are even more effective for controlling drying shrinkage [16]. Several theories could be used to explain the shrinkage reducing effects of SRAs from different points of view; however, the consensus is that the shrinkage reducing effects of SRAs are due to the their ability to lower the surface tension of pore solutions in cementitious materials [17]. Moreover, SRAs are extremely beneficial for reducing the magnitude of capillary tension which can slow the release rate of pore solution while the concrete loses moisture (during semi-insulated curing or drying shrinkage processes) [18,19]. Many studies have demonstrated that the shrinkage reducing effect of SRAs is related to the environmental or internal relative humidity (RH). Thus, many studies have focused on the SRAs’ functions under varied humidity conditions [2,12,19,20,21,22]. For example, SRAs can reduce the sorptivity and wetting moisture diffusivity of cementitious materials by improving the viscosity of their pore solutions [18]. Furthermore, an early-age expansion has been observed which was due to the increase in portlandite oversaturation and its crystallization within the pore solution when adding SRAs to cements [23]. Nevertheless, a critical threshold concentration of SRAs for reducing the surface tension of pore fluid has been observed, and the addition of SRA that exceeds the threshold concentration has resulted in the accumulation of surfactant molecules within the bulk solution rather than at the solution–air interface [19].

While researchers do not agree on how important the role of SRAs is for cement hydration, they acknowledge that the delay and regulation effects of SRAs change the pore structure of concrete [24,25]. The addition of SRAs to normal concrete with high w/c ratios (0.43 and 0.65) increased the total porosity and decreased the volume of pores with diameters ranging from 300 to 1000 nm [14]. Several microvoids in the range 10–20 μm were determined to be introduced in the microstructure of high-performance concrete featuring a higher w/c ratio (0.30). By lowering the water diffusion rate across the interface between the old (without SRA) and new (with SRA) concrete, the properties of the interface became more durable and the interface was able to endure moisture attacks.

Previously published papers indicated that, under different humidity conditions, the mechanical or durability properties of cementitious materials are strengthened or weakened by the addition of SRAs, and these effects are either directly or indirectly caused by the following factors: (1) the varied interfacial energy [26], surface area, and water demand [27] of the hydration products; (2) the pH value, polarity, and alkali contents of the pore solution [17,19,26]; and (3) a “diffusion barrier” [11] which is adsorbed on the surface of unhydrated cement particles. The mechanisms of action of SRAs have raised significant concerns in the research field; however, only early (28 days or less) physical changes [23,25,28,29] have been frequently discussed rather than long-term [14] (>28 days) chemical interactions.

This study mainly aimed to investigate the effects of two different SRAs on the volume stability of cement mortars under a designed alternating wet and dry schedule, accompanied by alternating temperature conditions. Mechanical and electrical sensors have been used for many years for the investigation of creep and shrinkage of cementitious materials [28]. However, these methods use point sensors which are installed on the surface of concrete and are difficult for laboratory use and suffer from long-term instability and fluctuation due to the fact of environmental effects and temperature [29].

The volume changes image processing system with acquisition, transmission and display was realized based on the complementary metal oxide semiconductor (CMOS) image sensor accordingly. The measurement involved the edge enhancement of input images, the reading of the intensities of each pixel (picture element) and the determinations of the outline of a specimen and the distribution of diameter changes [30]. Moreover, the representative characteristics of the hydration products and microstructure of cement mortars with and without added SRAs were determined using X-ray diffraction (XRD), scanning electron microscopy (SEM), mercury intrusion porosimetry (MIP) and nuclear magnetic resonance (NMR) techniques. The research results provided a better description of the shrinkage and swelling behaviour of cement mortars containing SRAs under wet–dry curing conditions and offered an effective approach for characterizing the distinction between the development of the gel structure of the hydration products with and without added SRAs.

## 2. Experimental

### 2.1. Materials and Mortar Composition

Portland cement (52.5 MPa strength grade) was obtained from Beijing Xingfa Cement Co., Ltd., China. The detailed chemical and mineral compositions of the Portland cement are summarised in Table 1 and were obtained using X-ray fluorescence spectrometry and XRD, respectively. The physical and mechanical properties of the cement are listed in Table 2.

Eclipse shrinkage-reducing agent (SRA-G) obtained from Grace China Chemical Building Materials Co., Ltd. and JM-SRA shrinkage-reducing agent (SRA-N) procured from Jiangsu Bot New Materials Co., Ltd. were used in this study, and their physicochemical properties and molecular compositions are listed in Table 3.

To improve the properties of fresh cement mortar, a powdery non-air entraining type high-concentration naphthalene superplasticizer (NSP) featuring the water reduction rate of 20% and conventional content of approximately 0.75% (by cementitious materials weight) was incorporated. The fine aggregate used in this study was China ISO standard sand produced by Xiamen Eszter Standard Sand Co. which was conformed to Chinese standard GB/T17671. The fineness modulus of the fine aggregate was 3.1. The mix proportion of cement mortar with SRA for the testing is listed in Table 4. The SRA-free specimen was used as a reference. For the mass loss test, the cement mortar specimens were demoulded after final setting and stored in water at 20 °C until 24 h; after that, the mass loss during the primary drying process (see Section 2.4) were tested. In this paper, the mass losses of three samples were tested at room temperature.

### 2.2. Axial Length Change Monitoring Using CMOS Image Sensor

**Figure 1:**
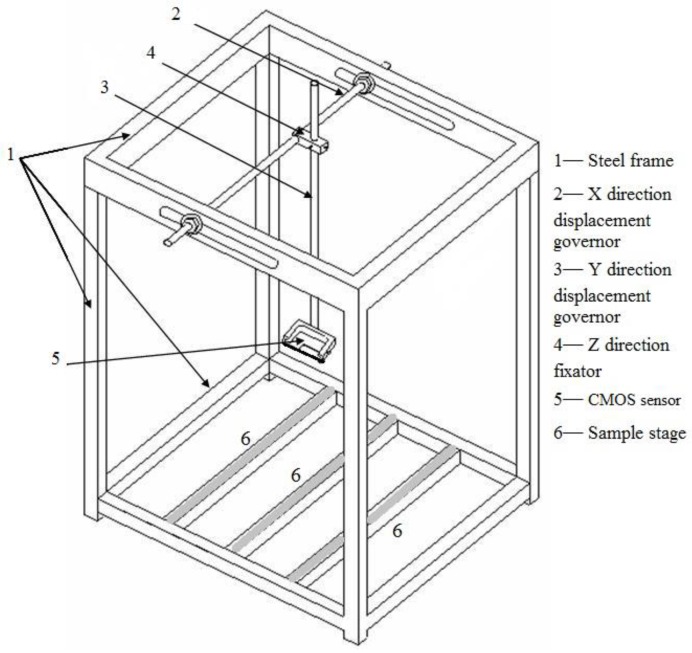
Schematic of geometry of unrestrained axial length change testing equipment.

This study mainly focused on the unrestrained axial length change (UALC) of hardened cement mortar which is caused by either shrink or swelling. The length changes were monitored using a 7-megapixel CMOS image sensor instead of the length comparator used in ASTM C157. The CMOS image sensor used for picture capturing could be moved simultaneously with the X–Y direction displacement governor (parts 2 and 3 in Figure 1) which was placed on the top of the steel frame (part 1 in Figure 1) with a precision of 0.1 μm. The distance detector was focused on the Z direction fixator (part 4 in Figure 1) which was 0.275 m from the three grey-coloured bottom stages (part 6 in Figure 1). Every 3 samples with 7 different mix proportions, as shown in Table 1, were tested and analysed in this study. Fresh cement mortar mixtures were poured into the mould after mixing and then mounted on the three fixed stages (part 6 in Figure 1) which were coated with sufficient lubricant oil to reduce friction, and therefore the setup was favourable for capturing repetitive images and ensured entirely steady sample positions.

**Figure 2:**
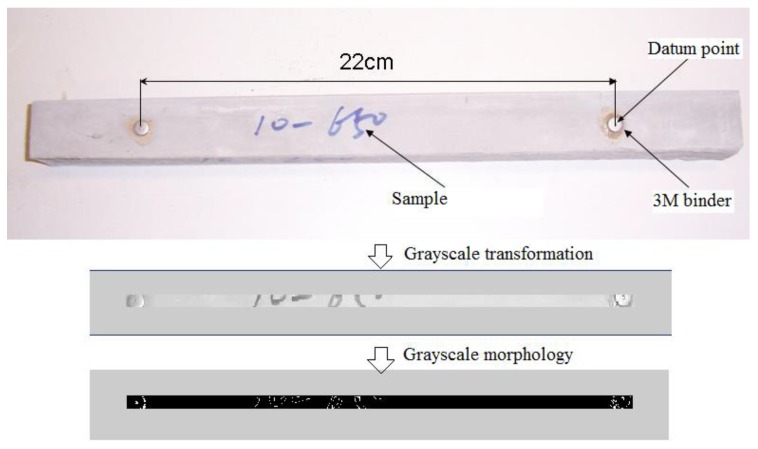
Schematic diagram of unrestrained axial length change measurements using digital image processing.

The length change was calculated using the digital image processing technology (DIPT) software developed by the researchers at the China Building Materials Academy, China. First, to enhance operation efficiency, grayscale processing was adopted to convert the colour images captured into grayscale ones (see Figure 2). Second, the featured points and edges of two datum points (220 mm distance) for the intensity and range images were acquired by analysing the average intensity and range variations of every three mortars with the same mix proportion. Lastly, both the initial and final lengths, the latter being caused by shrinking or swelling, were obtained using the DIPT software. The initial length was recorded just after the final settings were accomplished (several hours after moulding).

Accordingly, the UALC rate can be obtained using Equation (1):

(1) $$\varepsilon =\frac{L_{0}-L_{t}}{L_{0}}$$

where *ε* represents the UALC per unit length (με) of the specimen, *L*_0_ is the initial distance (mm) among the two datum points on the sample, and *L_t_* is the distance (mm) among the datum points after deformation which corresponded to the testing time (*t*).

### 2.3. Curing Conditions

#### 2.3.1. Hot–Dry Curing

Hot–dry conditions are known to be the most adverse simulated conditions for concrete experiencing drying shrinkage and cracking which represents a problem associated with the long-term volume stability of cementitious materials [27]. This study was conducted in the city of Beijing, North China, where the climate is hot–dry from July to September, the mean daytime temperature is elevated (approximately 35 °C) and the humidity level of the air is low (30% RH).

#### 2.3.2. Alternating Wet and Dry Exposure

Wet–dry cycling conditions are one of the primary factors leading to the decrease in the durability of concrete structures in natural climate. Therefore, to accurately simulate the effects of environmental exposure on the volume stability of cement-based materials, an organised dry–wet–dry exposure procedure was adopted as indicated in Table 5. All samples were cured under the curing conditions, as shown in Table 5, during the mass loss and volume change testing. For the volume tests, the samples were stored under these conditions together with the apparatus. For the mass loss tests, the samples were demoulded after 24 h curing in water (20 °C) and then were cured under the primary drying exposure conditions for 60 days.

### 2.4. Characterization of Liquid Evaporation and Microstructural Properties

The weight loss related to water evaporation during the drying process for the same SRA liquid or solid sample was measured using a digital scale. The scale was equipped with a data acquisition device which measured the weight loss of the 25 × 25 × 285 mm mortar samples at 1 h intervals. Three mortar samples were tested for each mixture. In addition, the evaporation of deionised water solutions (with and without SRA) were also one-sided dried and measured using identical mortar moulds.

After the entire dry–wet curing period (148 days), as shown in Table 5, the powdered samples (particles smaller than 80 μm) from three mortars with the same mix proportion were subjected to XRD analysis using a Bruker D8 Advance diffractometer in θ–θ configuration which utilised Cu Kα radiation (1.54 Å) under the fixed divergence slit size of 0.6°. To analyse the hydration products, the morphology and elemental composition of the cement mortars with (5% by cement weight) and without SRAs, which were aged for 148 days, were studied using SEM and energy-dispersive X-ray spectroscopy (EDS), utilizing a ZEISS MERLIN Compact field emission scanning electron microscope. The SEM/EDS samples were fabricated following the ASTM C1723 standard. The pore structures of cement mortars with and without SRA were evaluated using MIP (Autopore IV 9500, Micromeritics Instrument Ltd., Norcross, GA, USA). The NMR spectra were acquired using a Varian Infinity Plus 300 MHz spectrometer (Bruker Instrument Ltd., Stuttgart, Germany) equipped with ChemMagnetics style magic-angle spinning probes.

## 3. Results and Discussion

### 3.1. Evaporation of Solutions and Mortars

**Figure 3:**
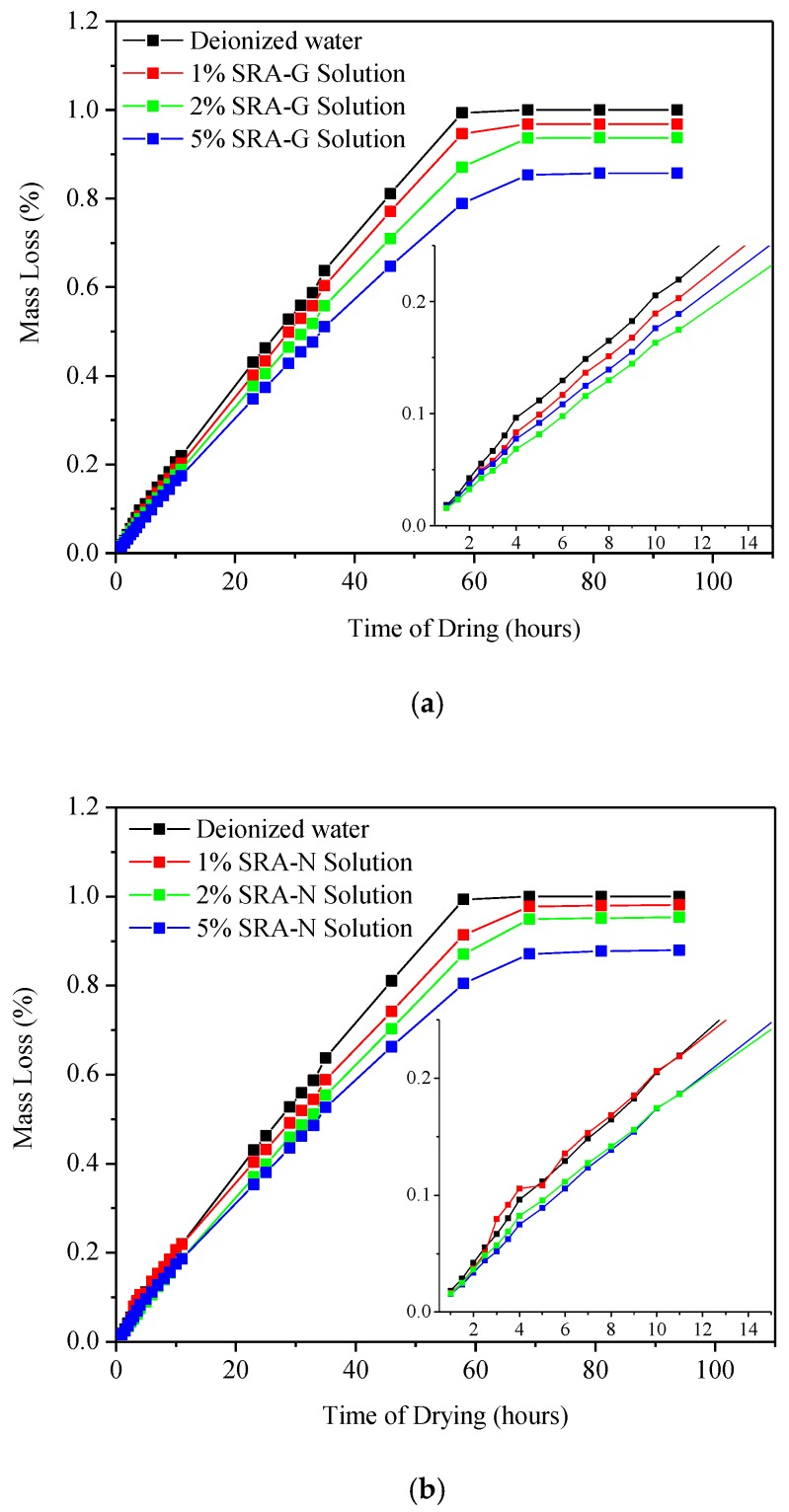
Evaporation performance of deionised water and shrinkage-reducing admixtures-incorporating solutions; (**a**) SRA-G; (**b**) SRA-N.

The evaporation performances were evaluated by comparing the weight loss of deionised water and SRA-blended liquids (see Figure 3). The weight loss was determined by placing each sample on a scale and recording the mass change owing to water evaporation per unit of weight at different aging times. The effect of the addition of SRA to water on the evaporation rate of deionised water appeared to be negligible during the first few hours [12]. Similar results were observed for the weight loss, but for extended testing periods. After 5 h of drying, the evaporation rates of the liquids containing SRAs were slightly lower than those of water for both SRAs at all dosages. The decrease in amplitude of the evaporation rate was directly proportional with the SRA dosage. Polyether and polyols are chemical compounds with the general formula HO[–(CH_2_)_m_O–]_n_–(CH_2_)_m_–OH [30]. After mixing them with deionised water, the SRA-enriched liquids were stable and macroscopically homogenous owing to the weak intermolecular attractive forces between the SRA molecules and water (which is a low polar molecule) [26]. However, the miscibility of the system decreased as the polymerization degree of SRA increased at elevated temperature or after extended periods (24 h in this study). Moreover, SRA-rich phases formed at the surface of the liquid [28], which partially hindered and slowed the evaporation of the liquid underneath. At the same time, a significant gap was observed between the equilibrium masses of the measured liquids even if evaporation equilibrium had been reached. This indicated that viscosity dominated the evaporation process when all other conditions were identical. Therefore, SRAs were still present in the systems when evaporation equilibrium was reached; however, the time required to reach evaporation equilibrium for the SRA-deionised water system decreased to an average of 20 h. As the viscosity of SRA-G was twice as high as that of SRA-N, the effect of SRA-G on reducing the evaporation rate of the liquid was more noticeable. Furthermore, the SRA-G-water system exhibited a smaller evaporation mass loss at equilibrium than the SRA-N-water one (see Figure 3). This assumption was in agreement with the results reported by Bentz [31], which stated that the addition of SRAs increased the viscosity of the liquid and, consequently, further reduced the evaporation rates of the liquids. Additionally, the effects were more noticeable as the SRA dosage increased.

**Figure 4:**
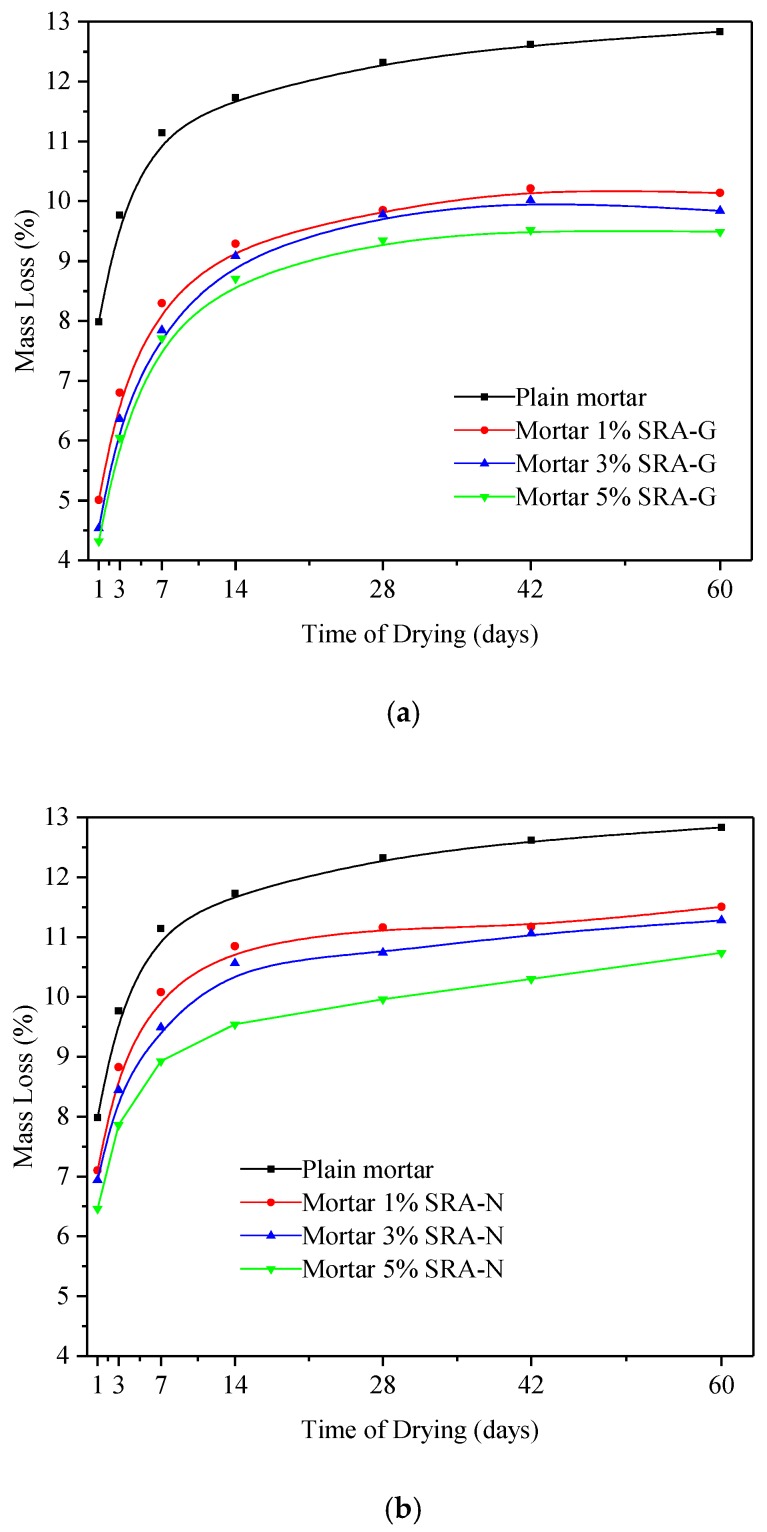
Mass loss of cement mortar (water-to-cement ratio of 0.3) under hot–dry conditions; (**a**) SRA-G; (**b**) SRA-N.

In addition to measuring the evaporation rate in solutions, the mass changes owing to surface evaporation was recorded for each mortar sample as presented in Figure 4. The abovementioned mass loss is an average value calculated on three tested data. The results indicated that the mass loss was higher during the early drying period, but it gradually decreased and became almost negligible during the late drying period for all samples. However, the incorporation of both SRAs decreased the overall mass loss and the decrease in mass was more even. It was determined that while both SRAs decreased the loss of water vapor, the effect of SRA-G was more evident. As the addition of SRAs led to the increase in viscosity, the liquid mass transportation from the interior toward the surface of the mortar became increasingly difficult. Although the rate of moisture loss during the early drying stages was different than the one during the later stages, the evaporation process began to stabilise at 28 days and stabilization eventually ended after 60 days. If the masses of the SARs themselves were taken into consideration, the final mass losses of the samples with the same w/c ratio were nearly the same as water evaporation reached equilibrium which indicated that the addition of SRAs only affected the rate of evaporation, and the effects of SRAs on the total amount of evaporable water was negligible.

### 3.2. Effect of SRAs on Shrinkage Properties under Dry and Hot Curing Conditions

**Figure 5:**
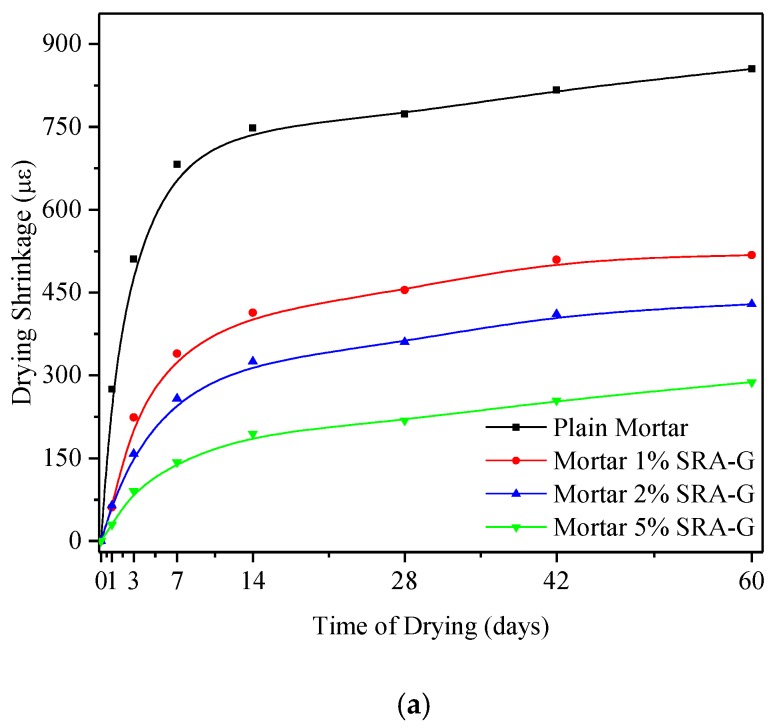

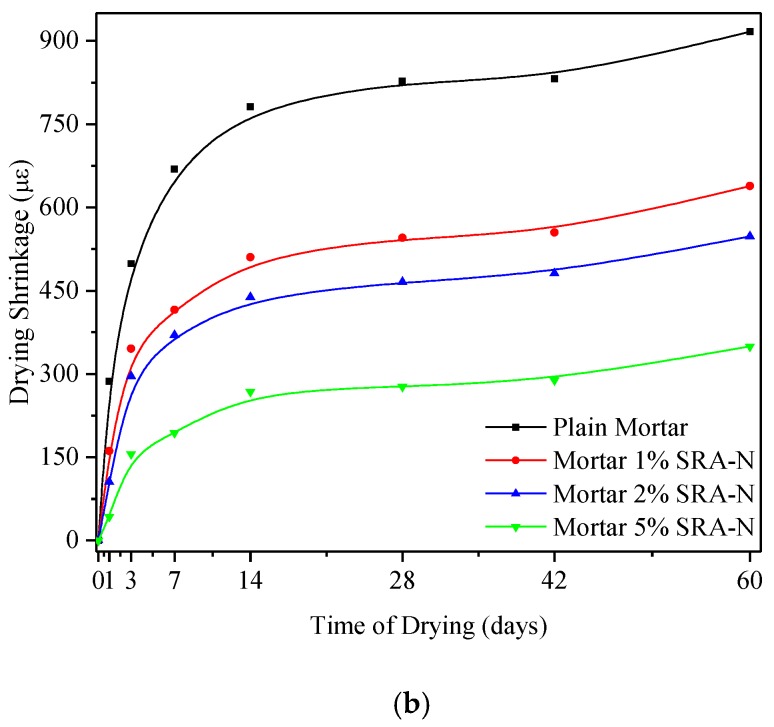
Shrinkage behaviour of cement mortars under hot–dry curing conditions; (**a**) SRA-G; (**b**) SRA-N.

Figure 5 illustrates the typical shrinkage performance of the samples subjected to hot–dry curing conditions for the first 60 days. The results indicated that SRAs were able to more efficiently reduce the shrinkage during the first 7 days, and their positive effect on shrinkage increased as their content increased but decreased with time. When analysing the effect of SRAs on the final drying shrinkage within 60 days, it was determined that the drying shrinkage of all samples stabilized after 7 days. The maximum shrinkage value of the reference sample was as high as 275 με after the first day and decreased to less than 30 με by the addition of 5% SRA (see Figure 5a). The maximum drying shrinkage value of the reference sample was 956 με after 60 days but was only 288 με for the SRA-incorporating mortar samples. The result shows that the effect of SRA on shrinkage restriction was obvious at the early stage, but it was not so obvious at late stage. This finding was consistent with those reported in previously published papers for high RH conditions which indicated that the SRA-incorporating mortars dried more slowly at high RH (liquid diffusion) during the early aging stage. However, this trend could be reduced at lower RH owing to the over drying that could occur when vapor diffusion prevails inside samples [17].

For the hot–dry curing conditions selected herein, the decrease in the shrinkage value of the SRA-G-incorporating mortar was superior compared with those of other SRA-incorporating samples. The SRAs featuring low surface tension and high viscosity values can easier alter the properties of the pore solution, and that could result in the decrease in the magnitude of the drying shrinkage of mortars [17].

### 3.3. Alternating Temperature and Humidity Conditions

#### 3.3.1. Re-Wet Curing

**Figure 6:**
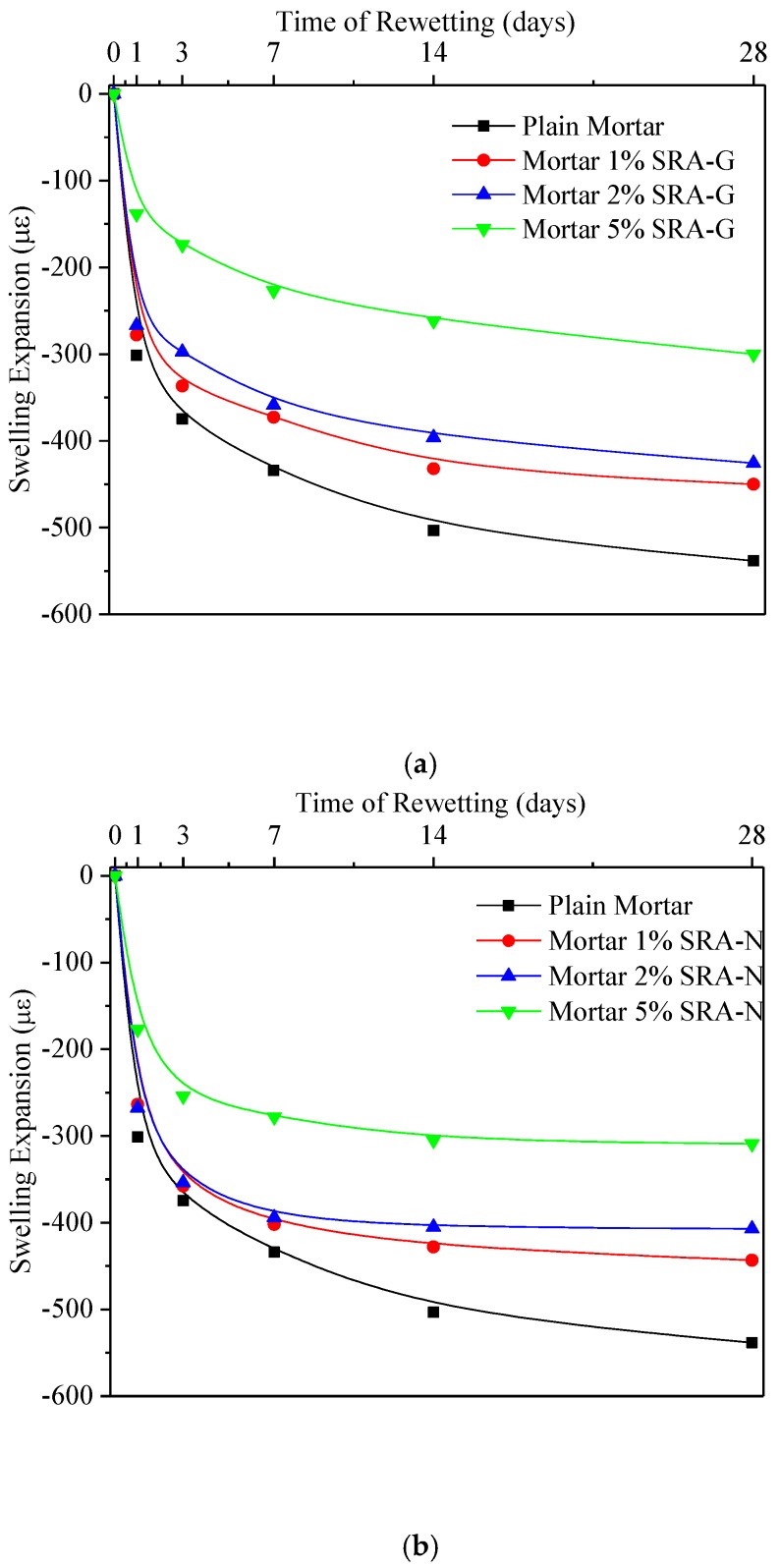
Length change of cement mortars under water curing conditions; (**a**) SRA-G; (**b**) SRA-N.

The length changes of rewetted mortar samples are presented in Figure 6. After 60 days of hot drying, as described in Section 3.2, followed by 100% RH wet curing, mortar samples inevitably exhibited wet swelling. Saint et al. [32] also reported slight swelling in their studies on the effect of SRAs on cement mortars. This indicated that such micro-expansion could be very effective at refining the long-term shrinkage of cement mortars. Subsequently, some researchers have pointed out that the addition of SRAs would increase the saturation level of dissolved calcium oxyhydroxide crystals in the pore solution of the cement mortar, which would result in the slight expansion of the cement-based materials [26] very early during the aging process.

Although some differences were observed, after the initial drying stage, all samples tended to rapidly undergo wet swelling when placed back in the relatively high humidity environment. For example, the length expansion of plain mortar was as high as 330 με in one day, which represented nearly half of its 28 days length expansion. The length expansion of the SRA-incorporating samples in the axial direction, however, was relatively small on the first day. Optimised mortar samples presented reductions of approximately 46% and 69% in swelling expansion compared with plain mortar. The length increase caused by swelling expansion slowed down after three days, which indicated the lack of sustained absorption momentum. Similar to the “late drying shrinkage” mentioned above, the effect of SRAs on the later swelling stage was not significant for the SRA-incorporating samples. The decrease in swelling of mortars is mainly due to the decrease in both penetration depth and water absorption rate with increasing SRA dosage [31].

#### 3.3.2. Secondary Drying

**Figure 7:**
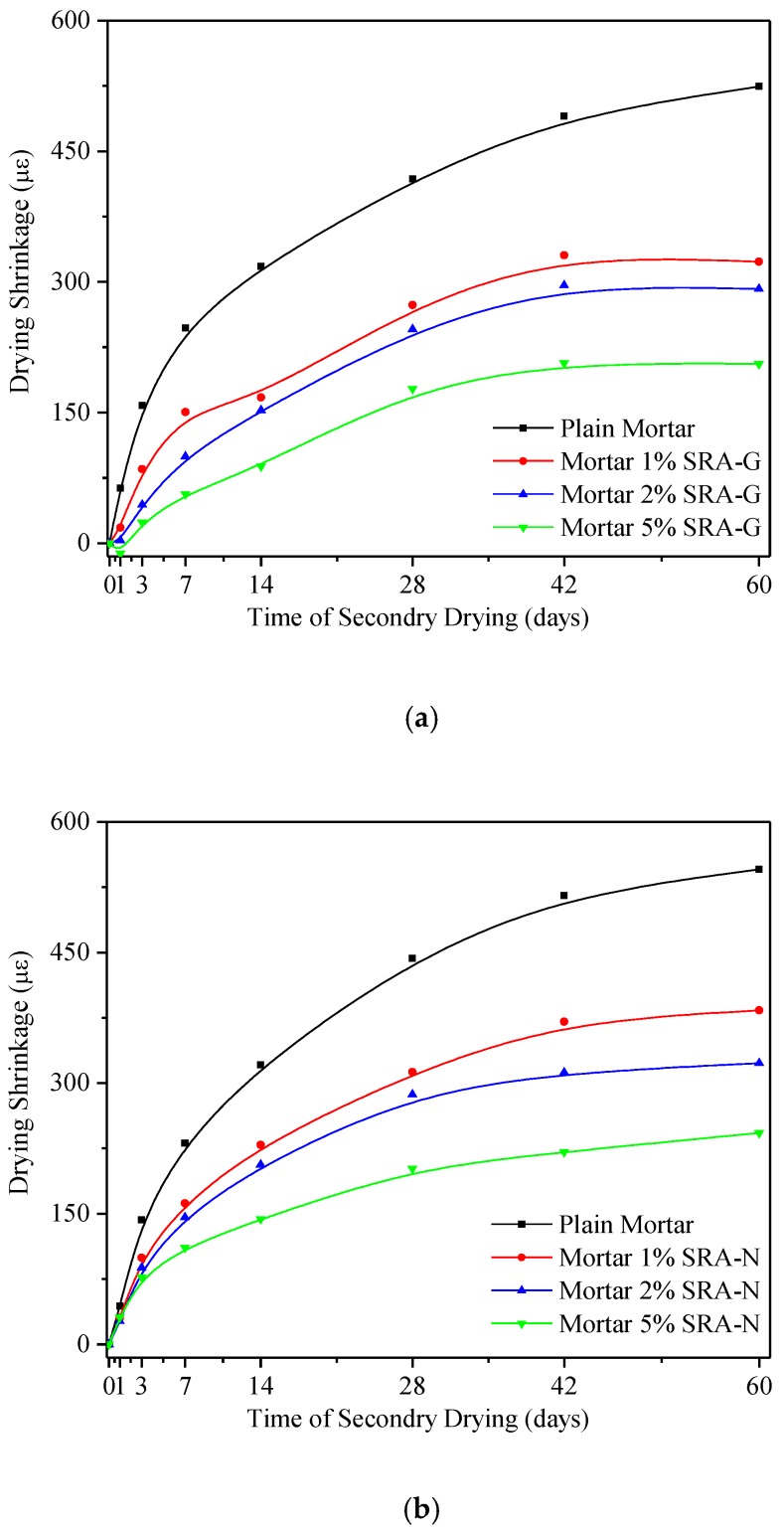
Secondary shrinkage performance of cement mortars (**a**) SRA-G; (**b**) SRA-N.

Figure 7 presents the 60 days shrinkage performance of cement mortars after 28 days of rewetting. Compared with the drying shrinkage behaviour during the first 60 days, illustrated in Figure 3, the amplitude of the length contraction for different samples under secondary drying conditions decreased by different percentages. The first-time shrinkage of the plain mortar sample decreased to approximately 1000 με, but it was merely 600 με at the end of the secondary drying process for the same sample which represented a decrease of approximately 40%.

The results obtained for the primary and secondary shrinkage behaviour indicated that the effects of SRAs on controlling the shrinkage decreased from the early (before 7 days) to the middle (7 to 28 days) and late (after 28 days) stages of treatment. This indicated that the development tendency and main characteristics of the early volume stability of cement mortar could be effectively identified by using the 7 days shrinkage performance instead of analysing the curve for the entire process (60 days). Thus, to intuitively evaluate the early age effect of the SRAs on the volume stability of hardened cement, the seven days axial length change factor, *K*_7_, was first put forward:

(2) $$K_{7}=\left|\frac{\varepsilon_{R}}{\varepsilon_{s}}\right|.$$

where *ε*_R_ and *ε*_s_ are the axial length change per unit of length (με) of the reference plain mortar and SRA-incorporating mortar at seven days, respectively. Accordingly, the *K*_7_ values calculated using the primary and secondary shrinkage values are listed in Table 6.

Comparing the shrinkage effectiveness of different SRA-incorporating samples, it was concluded that the samples doped with SRA-G exhibited higher *K*_7_ values than those doped with SRA-N for both drying processes (see Table 5). The higher *K*_7_ values of the SRA-G-doped mortars indicated their superior shrinkage reduction capacity under drying conditions. However, the primary and later secondary shrinkage reduction abilities of these samples were different. Generally, the shrinkage reduction ability of SRA-G exhibited a slight decrease during the secondary drying stage, but it was not significantly smaller than that recorded during the primary stage. By contrast, the *K*_7_ values of the SRA-N-doped samples decreased steeply and were reduced by half at the end of the secondary drying process. Therefore, both the primary and secondary shrinkage of the SRA-G-doped samples could be well confined by ensuring that the optimal amount of SRA-G was present in the pore solutions of these mortar samples at all times. When the samples were aged beyond a certain time period, the effect of SRA-N on reducing shrinkage was not significant. Previous studies have indicated that SRAs could delay or regulate cement hydration which could reorganise the microstructure development and affect the evolution of various performances of cement pastes [24,25]. In this study, the SRAs were able to prevent water from evaporating during the drying process and were, thus, able to potentially promote the hydration of cement or re-wet curing could promote the hydration of cement mortar while SARs could support or impair different hydration behaviour aspects during the water re-gaining process. The length changes of mortars illustrate the overall function and efficiency of each SRA, but idiographic analysis would be required to elucidate the microscale effects of SRAs.

### 3.4. Analysis of Macroscopic and Microscale Physical Properties

In this study, microscopic test methods, such as XRD, SEM, MIP and NMR, were adopted to qualitatively and quantitatively analyse the hydration behaviour of all samples, and the results provided certain theoretical support for evaluating the effects of SRAs on the volume stability of cement mortars under alternating temperature and humidity conditions.

**Figure 8:**
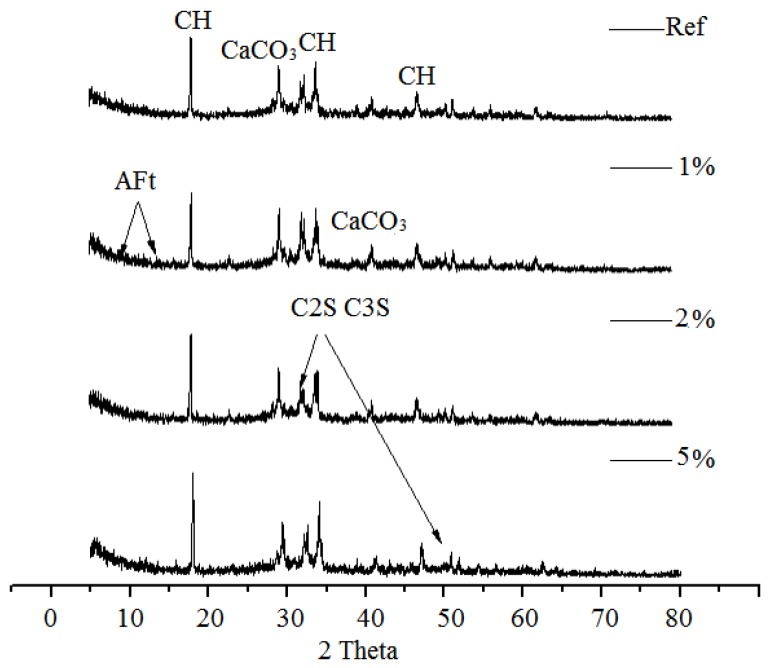
X-ray diffraction spectra of cement mortars containing different amounts of SRA-N.

**Figure 9:**
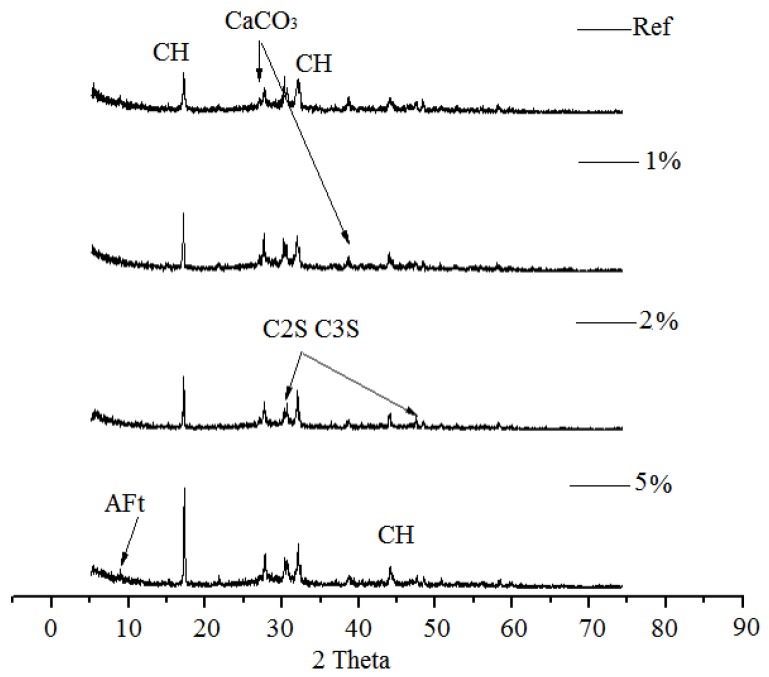
X-ray diffraction spectra of cement mortars containing different amounts of SRA-G.

Figure 8 and Figure 9 depict the XRD patterns of the hydration products after being subjected to alternating temperature and humidity conditions (60–28–60 days). The C_3_S and C_2_S diffraction peaks were identified in all samples even after 148 days of hydration which implied that the early age hot–dry environments might have hindered the hydration of cement to a different extent. However, the weak C_3_S and C_2_S peaks and the higher calcium hydroxide peaks were observed in all SRA-incorporating samples. This indicated that a superior hydration degree has been achieved during the drying process owing to the “water retention” effect of SRAs, which provided sufficient water for the cement to be hydrated [32]. For the SRA-incorporating mortars, more cement clinker would be involved in the hydration reaction and, thus, more calcium hydroxide would be generated during the alternating temperature and humidity conditions.

**Figure 10:**
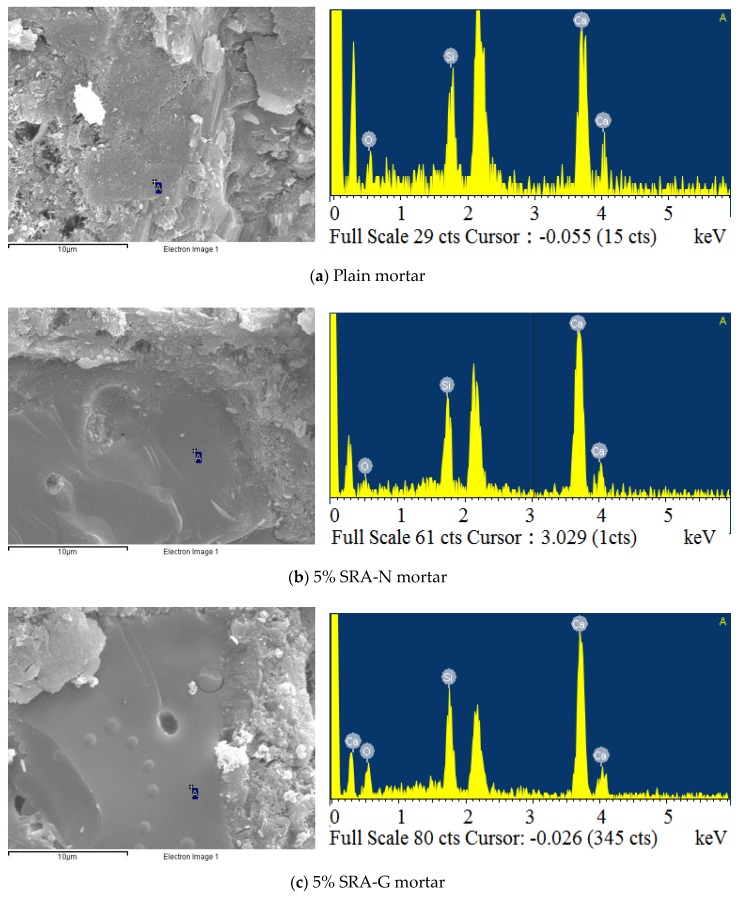
Scanning electron microscopy images and energy-dispersive X-ray spectroscopy elemental maps of different cement mortars.

Table 6 and Figure 10 illustrate the elemental maps and morphologies of the hydration products obtained using SEM and EDS analysis (A-point scanning) for three representative samples, including plain, 5% SRA-G, and 5% SRA-N mortars. As depicted in Figure 10a, the cement hydration products of plain mortar presented loose and uneven structural tightness which implied the lack of sufficient hydration products. Although some dents could be observed in the microstructure of the SRA-incorporating samples, most of the hydration products exhibited very compact structure and low apparent porosity (see Figure 10b).

Table 7 lists the EDS results obtained by scanning the “A” points in the SEM images of the samples. Compared with the hydration products of the plain mortar sample, the SRA-incorporating mortars presented higher Ca/Si ratios which indicated that the incorporation of the SRAs promoted the hydration of cement mortars under alternating temperature and humidity conditions. The SEM/EDS characterization results revealed that the improvement in stiffness was relative to the Ca/Si ratio of the hydration products and reflected in the microstructure of the calcium silicate hydrate (CSH) gel. In addition, the SEM images and EDS analysis indicated that all hydrated SAR-incorporating mortars comprised several regions of dense nano-sized CSH gels and gel pores which were dozens of nanometres in size. The percentage of gel pores was higher for the plain mortar sample.

To gather more detailed information on the pore structure of cement mortar, the MIP analysis results of the mortar after the entire dry–wet curing period (148 days) are summarised in Table 8. Compared with the plain mortar, the SRA-incorporating mortars exhibited significantly lower total porosity (the total porosity of the 5% SRA-G mortar decreased by approximately 6%). These findings contradicted the common assumptions on the redistribution of the porous structure by the addition of SRAs which indicated that SRAs should increase the total porosity [14] owing to the delayed hydration reactions they caused. The most critical cause responsible for the decrease in porosity was the “water retention” offset which occurred during the hot–dry curing periods. In the presence of sufficient water, SRAs should have played leading roles in delaying the hydration reaction rather than promoting water retention. At the same time, both smaller mean pore sizes and narrower pore distributions were observed for the plain mortar after 148 days of curing. The incorporated SRAs reduced the percentage of 3–50 nm pores but increased the percentage of 50–100 nm pores. Generally, the kinetics of evaporation are higher in larger pores, the larger capillary the pores, whereas the menisci in smaller pore remain at the surface [14]. However, the evaporation of water from larger pores would cause lower internal stress, and the “water rendition” of SRAs occurred during the drying process. This indicated that the beneficial effect on shrinkage reduction of the SRAs during hot–dry conditions was due primarily to the reconstruction of the porous structure, while the total porosity decreased. Moreover, the SRAs increased the mean pore size by inducing more 50–100 nm pores into the microstructure of the mortars. The traditional theory suggests that volume is regained when a specimen is re-wet. This fraction (swelling length change in this study) of the total drying shrinkage (primary shrinkage in this study) is, thus, called reversible or recoverable shrinkage. According to the Jennings classification methods of CSH gels [33], the low density (LD) CSH is less dense than the high density (HD) CSH which presents smaller pores that pressurised nitrogen cannot penetrate. Thus, HD CSH can be considered to belong to the restraining phase category, while LD CSH belongs to the shrinking phase category. The ratio of LD to HD CSH gels (LD/HD) in a cement paste would, thus, strongly affect its drying shrinkage, and the shrinkage of LD CSH could be irreversible. The total drying shrinkage was determined to be correlated best with the total pores volume, which could be measured using nitrogen (1–40 nm radius). The irreversible drying shrinkage correlated best with the small pores volume (1–4 nm radius), and the correlation coefficient for the latter was quite high which suggested a very strong relationship.

**Figure 11:**
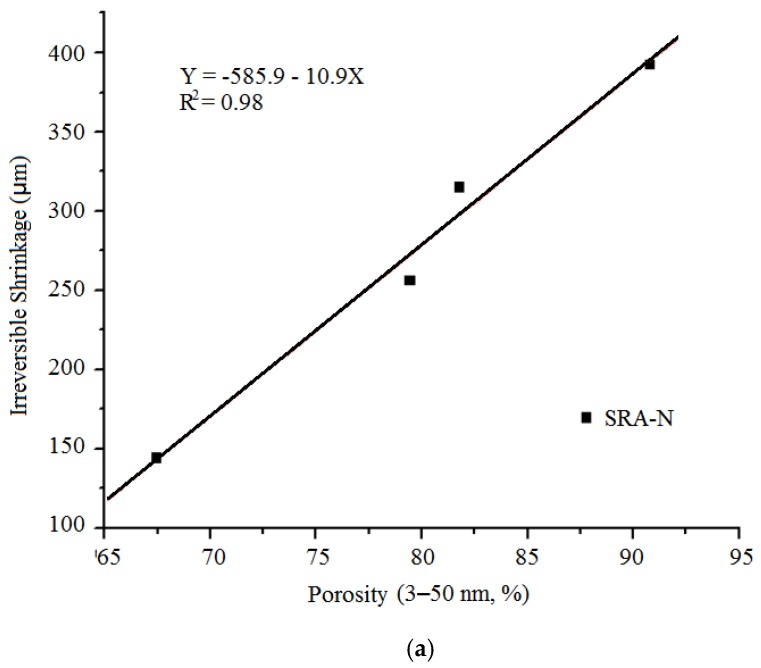

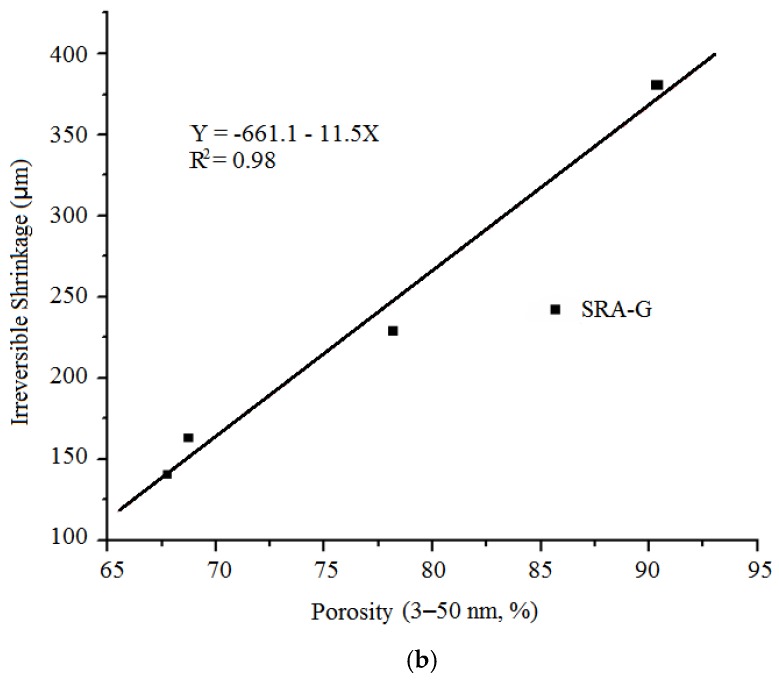
Relationship between secondary shrinkage and volume of 3–50 nm pores. (**a**) SRA-N; (**b**) SRA-G.

Reversible shrinkage depends on the quantity of CSH present, as determined by the degree of hydration, for a given w/c ratio. To verify the hypothesised relationship between the volume of the 3–50 nm pores and further (secondary) shrinkage, the plots for the strongest relationships between pore volumes and secondary shrinkage are illustrated in Figure 11. During the rewetting process, the volume of capillary pores (larger than 50 nm) was first recovered but without causing apparent length changes, while the volume of the HD CSH (less than 4 nm) phase was not recovered under atmospheric pressure conditions. The strong correlation between secondary shrinkage and volume of typical pores (3–50 nm radius) suggested that evaporation from such pores commonly occurred during the drying process.

**Figure 12:**
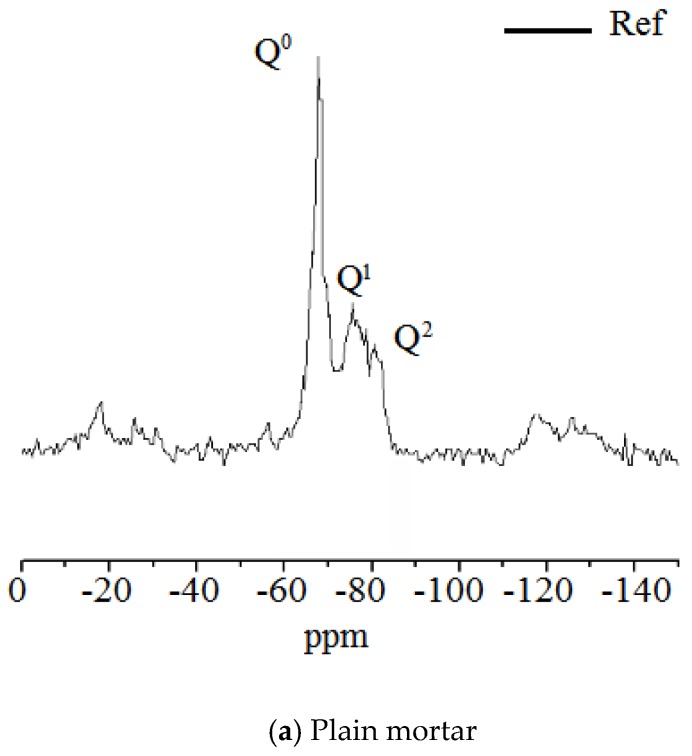

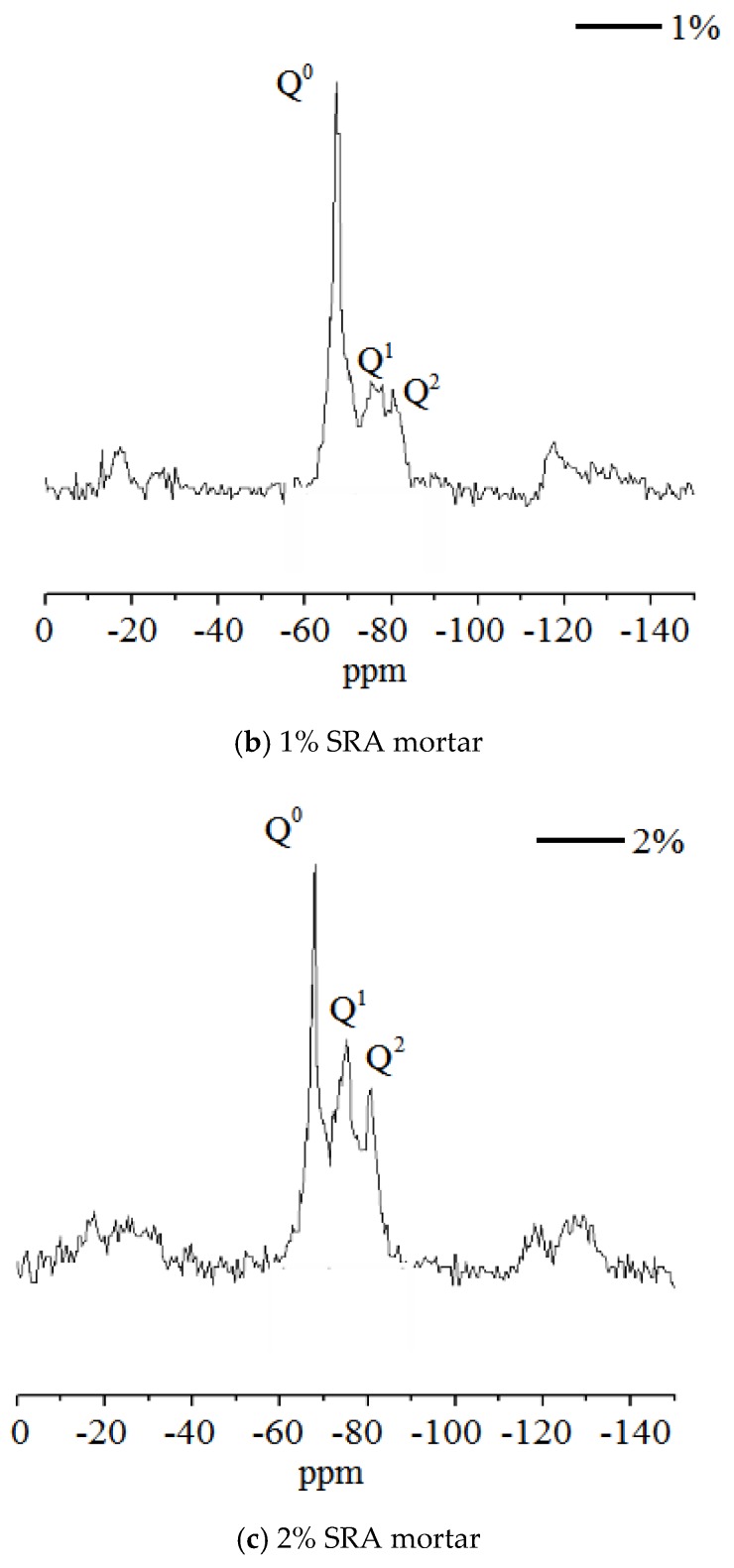

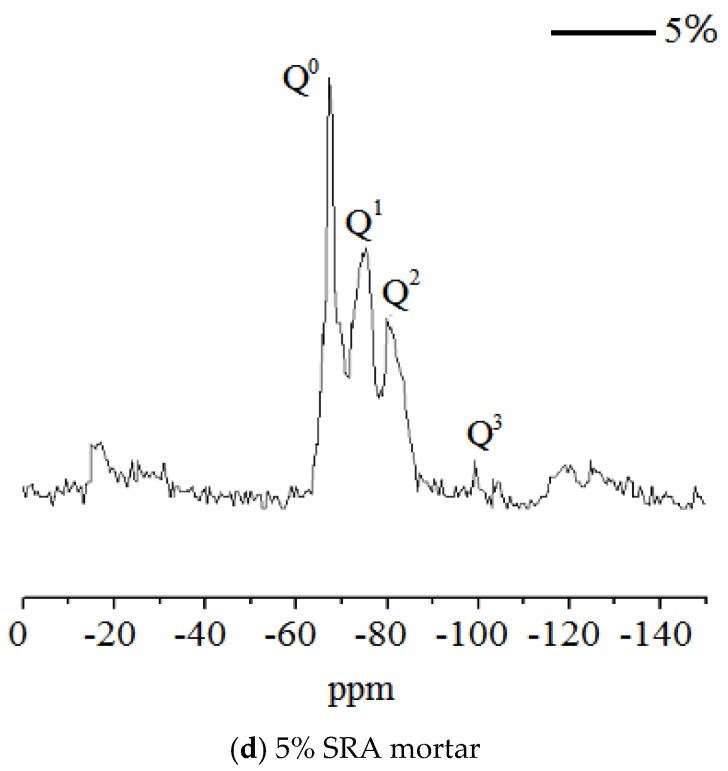
^29^Si nuclear magnetic resonance spectra of cement mortars containing different SRA-G dosages.

Figure 12 illustrates the ^29^Si NMR results of SRA-G-incorporating cement mortar. In the NMR spectrum, Q^0^ represents orthosilicic acid, an isolated siloxane tetrahedron, which is an unhydrated clinker, while Q^1^, Q^2^ and Q^3^ indicate one, two and three attached silicon tetrahedra, respectively, and Q4 is a three-dimensional grid structure connected with four silicon tetrahedra. As presented in Figure 12a, only two polymerization states of the CSH gel were identified which were considered to be Q^1^ and Q^2^. Moreover, the widths of both the Q^1^ and Q^2^ peaks increased as the SRA-G dosage increased. In fact, the peak shape of Q^2^ became more prominent which indicated that more cement was involved in the hydration process and more hydration products were converted from the oligomerised to the higher polymerised state.

The higher polymerization state, Q^3^, was observed when 5% SRA-G was incorporated into the mortar. The irreversible shrinkage of cement mortar was closely related to the degree of polymerization of the CSH gel [33]. Therefore, SRAs reduced the amount of irreversible shrinkage by increasing the hydration degree of cement mortar under alternating temperature and humidity conditions which converted the CSH gel into a highly polymeric structure and, consequently, increased the volume stability of the cement mortar.

## 4. Conclusions

This paper introduced an inexpensive, non-destructive and quantitative measurement of unharding paste length change. With this method, the length change during hydration and the wet–drying process can be monitored by a self-developed CMOS sensor. Non-destructive measurements of length change can be used to identify the effect of SRA on shrinkage of cementitious materials. Microstructural observations using XRD, SEM, MIP and NMR methods were performed to elucidate the role of SRAs on the volume stability of cement mortars. The following conclusions were drawn:

1. Not only did the SRAs significantly inhibit the shrinkage of cement mortar, but the decrease in shrinkage increased as the SRA dosages increased. The addition of SRAs to mortars moderated the liquid evaporation process but only affected the water loss process as a whole and exhibited little effect on the total amount of water that could be evaporated;
2. Although SRAs present certain inhibitory effects on wet swelling at different ages, this inhibition generally occurred in the early stages of aging. Owing to their “water retention” effect, the SRAs increased the Ca/Si ratio and hydration degree of cement mortars under alternating temperature and humidity conditions;
3. Secondary shrinkage in cement mortar was closely related to the volume of 3–50 nm pores, but total shrinkage could be confined when CSH gel achieved a higher polymerization state by incorporating high dosages of SRAs into mortars.

## Figures and Tables

**Table 1:** Chemical and mineral composition of cement (%).

SiO_2_	Al_2_O_3_	Fe_2_O_3_	CaO	MgO	Loss on Ignition	f-CaO	C_3_S	C_2_S	C_3_A	C_4_AF
21.45	4.08	2.41	63.71	2.29	1.99	0.87	58.29	21.39	7.30	6.98

**Table 2:** Physical and mechanical properties of cement.

45 μm Sieve Residual (%)	Blaine Specific Surface Area (m^2^/kg)	Setting Time (h:min)		Flexural Strength (MPa)		Compressive Strength (MPa)	
		Initial Setting	Final Setting	3 d	28 d	3 d	28 d
1.2	331	3:17	4:10	5.6	9.4	28.1	57.7

**Table 3:** Properties of shrinkage-reducing agents (SRAs).

SRA	Molecular Composition	Liquid Appearance	Density (g/mL)	Viscosity (Pa·s)	Surface Tension (mN/m)	Recommended Dosage (wt. %)
SRA-G	Polyether	Light yellow	1.04	100	32.5	2–3
SRA-N	Polyol	Clear	1.02	50	41.7	1–2

**Table 4:** Mix proportion of cement mortar for mass loss and volume change testing.

Number	Cement (kg/m^3^)	Water (kg/m^3^)	W/C	Sand (kg/m^3^)	Additives	
					NSP (%)	SRA (%)
Ref	450	135	0.30	1350	0.75	0
N-1	450	135	0.30	1350	0.75	1
N-2	450	135	0.30	1350	0.75	2
N-5	450	135	0.30	1350	0.75	5
G-1	450	135	0.30	1350	0.75	1
G-2	450	135	0.30	1350	0.75	2
G-5	450	135	0.30	1350	0.75	5

**Table 5:** Alternating wet and dry exposure procedure parameters.

Stage	Primary Drying	Rewetting	Secondary Drying
Condition			
Humidity (%)	30	100	30
Temperature (°C)	35	20	35
Curing period (days)	60	28	60

**Table 6:** Seven days axial length change factor, *K*_7_, values of mortar samples undergoing primary and secondary drying.

	SRA-G (%)				SRA-N (%)			
	0	1	2	5	0	1	2	5
Primary drying	1.00	2.01	2.64	4.77	1.00	1.61	2.49	4.38
Secondary drying	1.00	1.43	1.81	3.45	1.00	1.24	1.64	2.19

**Table 7:** Energy-dispersive X-ray spectroscopy analysis of mortars with and without shrinkage-reducing admixtures.

	Plain Mortar (Reference)			5% SRA-N Mortar			5% SRA-G Mortar		
	wt. %	at. %	Ca/Si	wt. %	at. %	Ca/Si	wt. %	at. %	Ca/Si
Si	20.48	17.40	1.4	15.96	14.02	2.1	13.73	12.26	2.6
Ca	40.16	23.90		47.06	28.97		50.48	31.61	
O	39.36	58.70		36.97	57.01		35.79	56.13	
Totals	100.00	100.00		100.00	100.00		100.00	100.00	

**Table 8:** Pore size distribution of cement mortars.

Code	Mean Pore Size (nm)	Porosity (%)	Pore Size Distribution (nm)				
			3–50	50–100	100–200	200–500	>500
Plain mortar	17.3	26.5	90.80	1.87	0.35	0.48	4.84
N-1	18.8	23.9	81.79	5.87	0.96	1.14	5.52
N-2	20.8	21.0	79.46	8.70	1.02	0.74	4.16
N-5	24.8	21.7	67.48	10.43	1.97	2.23	5.70
G-1	17.9	25.5	76.16	6.04	0.63	0.86	5.02
G-2	23.8	22	64.72	18.73	0.78	1.39	5.40
G-5	24.3	20.7	63.57	20.83	0.87	1.38	4.28
